# Clinicopathological Features and Disease Outcome in Breast Cancer Patients with Hormonal Receptor Discordance between Core Needle Biopsy and Following Surgical Sample

**DOI:** 10.1245/s10434-019-07480-y

**Published:** 2019-05-29

**Authors:** Siji Zhu, Jiayi Wu, Ou Huang, Jianrong He, Li Zhu, Yafen Li, Weiguo Chen, Xiaochun Fei, Xiaosong Chen, Kunwei Shen

**Affiliations:** 1 Comprehensive Breast Health Center, Ruijin Hospital, Shanghai Jiaotong University School of Medicine, Shanghai, China; 2 Department of Pathology, Ruijin Hospital, Shanghai Jiaotong University School of Medicine, Shanghai, China

## Abstract

**Background:**

There are limited data about how to manage patients with discordant hormonal receptor (HR) status between core needle biopsy (CNB) and following surgical sample (FSS). This study aimed to evaluate clinicopathological features and disease outcome for these HR discordance patients.

**Patients and Methods:**

Invasive breast cancer patients with paired HR between CNB and FSS were retrospectively analyzed, being classified into three groups: HR positive, HR negative, and HR discordance. Patient characteristics, treatment decisions, and disease outcome were compared among above groups.

**Results:**

A total of 1710 patients (1233 HR positive, 417 HR negative, and 60 HR discordance patients) were enrolled. Compared with the HR positive group, HR discordance patients were associated with more human epidermal growth factor receptor 2 positivity (*P* < 0.001) and higher Ki67 level (*P* = 0.001) tumors. The fraction of patients receiving adjuvant chemotherapy was 95.0% and 93.8% in the HR discordance or HR negative groups, much higher than in the HR positive group (66.7%, *P* < 0.001). Of 60 HR discordance patients, 34 (56.7%) received adjuvant endocrine therapy. The 5-year disease-free survival (DFS) rate was 90.4% for HR discordant patients, showing no statistical difference compared with HR positive (87.0%, *P* = 0.653) or HR negative (83.2%, *P* = 0.522) groups. For HR discordance patients, there was no difference in DFS between patients who received adjuvant endocrine therapy or not (*P* = 0.259).

**Conclusions:**

HR discordance patients had similar tumor characteristics, adjuvant chemotherapy treatment, and DFS compared with HR negative patients. The benefit of endocrine therapy in these HR discordance patients is uncertain and deserves further clinical evaluation.

**Electronic supplementary material:**

The online version of this article (10.1245/s10434-019-07480-y) contains supplementary material, which is available to authorized users.

Core needle biopsy (CNB), as an initial procedure in breast cancer (BC) diagnosis, is widely used and recommended to test tumor biomarkers, such as hormonal receptor (HR), human epidermal growth factor receptor 2 (HER2), and Ki67 status. The accuracy of CNB for breast cancer diagnosis is more than 95%.^1^^–^3 However, due to its relatively smaller sample size and tumor heterogeneity, biomarker assessment performed in CNB samples may be less reliable than in the following surgical sample (FSS). The accuracy rates for estrogen receptor (ER), progesterone receptor (PgR), and HER2 evaluation between CNB and FSS are reported as 61.7–99.0%, 61.5–97.1%, and 64.2–98.8%, respectively.^4^^–^6 In addition, for molecular subtype status analysis between CNB and FSS, a recent study showed that CNB was accurate in determining nonluminal molecular subtypes for invasive BC.^7^ The 2015 European Society for Medical Oncology (ESMO) guideline recommended that HR and HER2 status be first tested by CNB, which can be used to guide further systemic treatment.^8^ However, data regarding how to manage these patients with HR discordance tumors between CNB and FSS are lacking.

The aim of the current study is to investigate the clinicopathological features, adjuvant treatment choice, and disease outcome among patients with different HR status between CNB and FSS, which may guide our further clinical management.

## Patients and Methods

### Patients and Samples

Female patients who received operation for invasive breast cancer in Ruijin Hospital between January 2011 and December 2015 were included from the SJTU-BCDB breast cancer database. HR status was detected in both CNB and paired FSS. CNB was performed under ultrasound guidance, and at least four 14-gauge core biopsies were obtained for further pathological examination. Patients who received neoadjuvant treatment before surgery were excluded.

Core needle biopsy specimens were fixed immediately in adequate volume of 4% buffered formaldehyde and embedded in paraffin for histopathological analysis. A minimum fixation time of 6 h was ensured, according to American Society of Clinical Oncology/College of American Pathologists (ASCO/CAP) guidelines,^9^,^10^ prior to tissue processing and paraffin embedding. Lumpectomy specimens were incised into the tumors, the mastectomy specimens were cut into 1-cm-thick slices before fixation, and the time from tumor removal to fixation was within 1 h to comply. Sampled tissue blocks were fixed in adequate volume of 4% buffered formaldehyde then embedded in paraffin.

### Receptor Status Testing

Immunohistochemistry (IHC) was performed on formalin-fixed, paraffin-embedded tissue sections using a Ventana autostain system, BenchMark XT, for breast tumor specimens from CNB and FSS to evaluate receptor status. The cutoff value for ER positivity and PgR positivity was at least 1% positive tumor cells with nuclear staining.^9^ Discordance between the tumor hormone receptor profiles of CNB and FSS was considered when both HR assays were negative on one examination, and at least one HR assay was above 1% on the other examination. Patients were classified into the following groups according to HR status in CNB and FSS: HR positive (both HR + in CNB and FSS), HR negative (both HR– in CNB and FSS), and HR discordance (HR– in CNB and HR + in FSS, or HR + in CNB and HR– in FSS).

HER2 status was first examined by IHC using a 0–3+ score according to the ASCO/CAP guideline.^10^ Tumors with IHC HER2 2+ were further examined by fluorescence in situ hybridization (FISH), and HER2 positivity was defined as IHC HER2 3+ or FISH + . For Ki67 expression scoring, we first reviewed the cell distribution over the whole slice. If Ki67 expression was uniformly distributed over the entire slide, 500–2000 cells were chosen from different microscope views; otherwise, 2000 cells were equally counted in both hotspot and negative areas in the slide. Ki67 expression was scored as the percentage of positive invasive tumor cells with any nuclear staining and recorded as mean percentage of positive cells.

The following antibodies were used for IHC testing: ER (SP1, DAKO), PgR (PgR 636, Dako), HER2 (4B5, Roche), and Ki67 (MIB-1, Dako). All IHC and FISH analyses were reviewed by two pathologists of the Department of Pathology, Ruijin Hospital, Shanghai Jiaotong University School of Medicine.

### Follow-Up

All patients were followed up by outpatient visit or call every 3 months for the first 2 years after surgery, every 6 months between the 3rd and 5th years, then annually every year until death. Disease-free survival (DFS) was defined as the time period from the date of operation to the date of the following events: distant recurrence, locoregional recurrence, contralateral breast cancer, secondary nonbreast malignant tumors, and any cause of death. Overall survival (OS) was defined as the time period from the date of operation to the date of death by any cause.

### Statistical Analysis

Descriptive analysis was conducted to calculate the clinicopathological features and treatment choices. Concordance analysis of receptor status between CNB and FSS was performed by kappa test. *κ* value > 0.6, 0.4–0.6, 0.2–0.4, and < 0.2 were classified as good, moderate, fair, and poor agreement, respectively. Chi square test and multivariate logistic regression analysis were used to compare the distribution of characteristics among different HR status subtypes. The estimated 5-year DFS and OS were calculated by Kaplan–Meier analysis. Cox proportional hazards regression analysis was performed to examine the impact of clinicopathological features on disease outcomes. All *P* values were two-sided, with values less than 0.05 considered statistically significant. All statistical procedures were performed by using SPSS software, version 20.0 (SPSS Company, Chicago, IL).

## Results

### Patient Characteristics

**Fig. 1:**
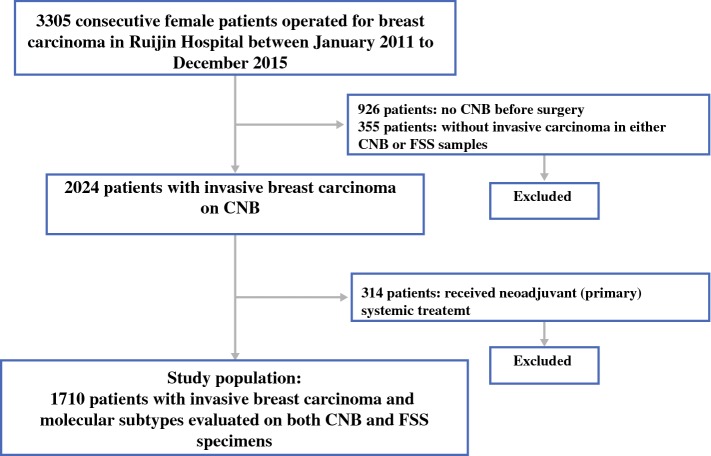
Identification of study population

Between January 2011 and December 2015, 3305 consecutive female breast cancer patients received surgery. A total of 1710 invasive breast cancer patients were included in this study after excluding patients according to the eligibility criteria (Fig. 1). The median age was 56 (23–95) years, and 1113 patients (65.1%) were postmenopausal. A total of 1206 patients (70.5%) received mastectomy, and 1003 patients (58.7%) had negative axillary lymph nodes (Table 1).

**Table 1:** Patient characteristics

Characteristics	No.	%
Age (years)	56 (23–95)	
≤ 50	559	32.7
> 50	1151	67.3
Menstrual status		
Peri/pre-menopause	597	34.9
Post-menopause	1113	65.1
Breast surgery type		
Lumpectomy	504	29.5
Mastectomy	1206	70.5
Tumor size		
≤ 2 cm	857	50.1
> 2 cm	848	49.6
NA	5	0.3
Axillary lymph node		
Negative	1003	58.7
Positive	702	41.0
NA	5	0.3

### Comparison of CNB with FSS for Receptor Status and Ki67

Expression rates of receptors and Ki67 between CNB and FSS are presented in Supplementary Table 1. In CNB samples, 1266 (74%) and 444 (26%) patients were classified as HR+ and HR−, respectively. Regarding FSS sample, HR positivity was 73.7% (1260 cases). Similarly, the positivity rate of ER was 73.8% and 73.2% in CNB and FSS, and PgR was 55.0% and 57.9% in CNB and FSS, respectively. There were 19.6% and 22.0% HER2 positive tumors in CNB and FSS. Differences for HR, ER, PgR, and HER2 status between CNB and FSS were not statistically significant (*P* > 0.05). The concordance rate between CNB and FSS was 96.5%, 96.5%, 91.1%, and 95.3% for HR, ER, PgR, and HER2, respectively (Supplementary Table 2). Median Ki67 was 15% for CNB and 20% for FSS, and mean Ki67 expression was 28.6% in FSS, higher than in the CNB samples (24.3%, *P* < 0.001), and the concordance rate for Ki67 expression level was 81.5% (Supplementary Tables 1 and 2).

### Tumor Characteristics Among Patients with Discordant HR Status

A total of 60 cases (3.5%) had discordant HR between CNB and FSS: 33 patients with HR CNB+/FSS− and 27 patients with HR CNB−/FSS+. There were 31 patients (14 with CNB−/FSS+, 17 with CNB+/FSS−) with low ER expression (ER expression less than 10% in CNB or FSS) (Supplementary Fig. 1).

There were 1233 and 417 cases in the HR positive and HR negative groups. Regarding tumor characteristics among groups with different HR status, no statistically significant difference was observed in terms of age, menstrual status, and axillary lymph node status. Tumor larger than 2.0 cm was found in 46.9%, 56.4%, and 58.3% patients with HR positive, HR negative, and HR discordance tumor, respectively (*P* = 0.001; Table 2). The rate of high Ki67 expression was 58.5%, 88.5%, and 91.7% in the HR positive, HR negative, and HR discordance patients (*P* < 0.001). There were 40.8% patients in the HR negative group and 53.3% in the HR discordance group with HER2 positive disease, which was higher than patients in the HR positive group (14.4%, *P* < 0.001). Moreover, pathological type (*P* = 0.011) and histological grade (*P* < 0.001) were statistically significantly different among these three groups.

**Table 2:** Univariate analysis of tumor characteristics according to hormonal receptor Status

Characteristics	HR+ (*n* = 1233)		HR- (*n* = 417)		HR discordance (*n* = 60)		*χ* ^2^	*P*
Age							1.315	0.518
≤ 50	409	33.2%	128	30.7%	22	36.7%		
> 50	824	66.8%	289	69.3%	38	63.3%		
Menstrual status							2.679	0.262
Peri/pre-menopause	440	35.7%	133	31.9%	24	40.0%		
Peri/pre-menopause	793	64.3%	284	68.1%	36	60.0%		
Tumor size							12.965	0.002
≤ 2 cm	651	52.8%	181	43.4%	25	41.7%		
> 2	578	46.9%	235	56.4%	35	58.3%		
NA	4	0.3%	1	0.2%	0	0%		
Axillary lymph node							3.082	0.214
Negative	707	57.3%	259	62.1%	37	61.7%		
Positive	522	42.4%	157	37.6%	23	38.3%		
NA	4	0.3%	1	0.2%	0	0%		
Pathological type							13.054	0.011
IDC	1099	89.1%	392	94.0%	55	91.7%		
ILC	43	3.5%	2	0.5%	2	3.3%		
Others	91	7.4%	23	5.5%	3	5.0%		
Histological grade							185.869	< 0.001
I	40	3.2%	0	0%	1	1.7%		
II	644	52.3%	89	21.3%	15	25%		
III	434	35.2%	303	72.7%	40	66.7%		
NA	114	9.3%	25	6%	4	6.7%		
HER2							159.821	< 0.001
Positive	178	14.4%	170	40.8%	32	53.3%		
Negative	1055	85.6%	247	59.2%	28	46.7%		
Ki67							96.651	< 0.001
< 14%	512	41.5%	48	11.5%	5	8.3%		
≥ 14%	721	58.5%	369	88.5%	55	91.7%		

*HR* hormonal receptor, *HER2* human epidermal growth factor receptor-2, *IDC* invasive ductal carcinoma, *ILC* invasive lobular carcinoma

Multivariate analysis demonstrated that HER2 and Ki67 status were statistically different between the HR discordance and positive groups (Table 3). Compared with patients in the HR positive group, patients in the HR discordance group had higher Ki67 expression tumors [odds ratio (OR) 5.009, 95% confidence interval (CI) 1.944–12.908, *P* = 0.001] and more HER2 positive disease (OR 4.727, 95% CI 2.737–8.164, *P* < 0.001). There was no statistically significant difference in terms of those tumor characteristics between the HR discordance and HR negative groups.

**Table 3:** Multivariate analysis of tumor characteristics according to hormonal receptor statusa

Characteristics	HR positive (*n* = 1233)			HR discordance (*n* = 60)			*P* value
	OR	95% CI	*P*	OR	95% CI	*P*	
Tumor size							0.883
≤2 cm	1.055	0.823–1.852	0.673	0.961	0.549–1.681	0.889	
>2 cm	1			1			
Pathological type							0.025
ILC	9.863	1.838–52.923	0.008	15.844	1.052–238.535	0.046	
Others	1.540	0.629–3.771	0.345	1.830	0.268–12.485	0.072	
IDC	1			1			
Histological grade							< 0.001
I	∞	169,771,835–∞	< 0.001	∞	∞-∞	< 0.001	
II	3.327	2.497–4.433	< 0.001	1.495	0.771–2.899	0.234	
III	1			1			
HER2							< 0.001
Positive	0.366	0.280–0.480	< 0.001	1.698	0.969–2.976	0.064	
Negative	1			1			
Ki67							< 0.001
< 14%	2.549	1.785–3.639	< 0.001	0.576	0.200–1.662	0.308	
≥ 14%	1			1			

*HR* hormonal receptor, *HER2* human epidermal growth factor receptor-2, *IDC* invasive ductal carcinoma, *ILC* invasive lobular carcinoma

^a^The reference category for subtype characteristics is HR negative group

### Hormonal Receptor Discordance and Adjuvant Systemic Therapy

**Fig. 2:**
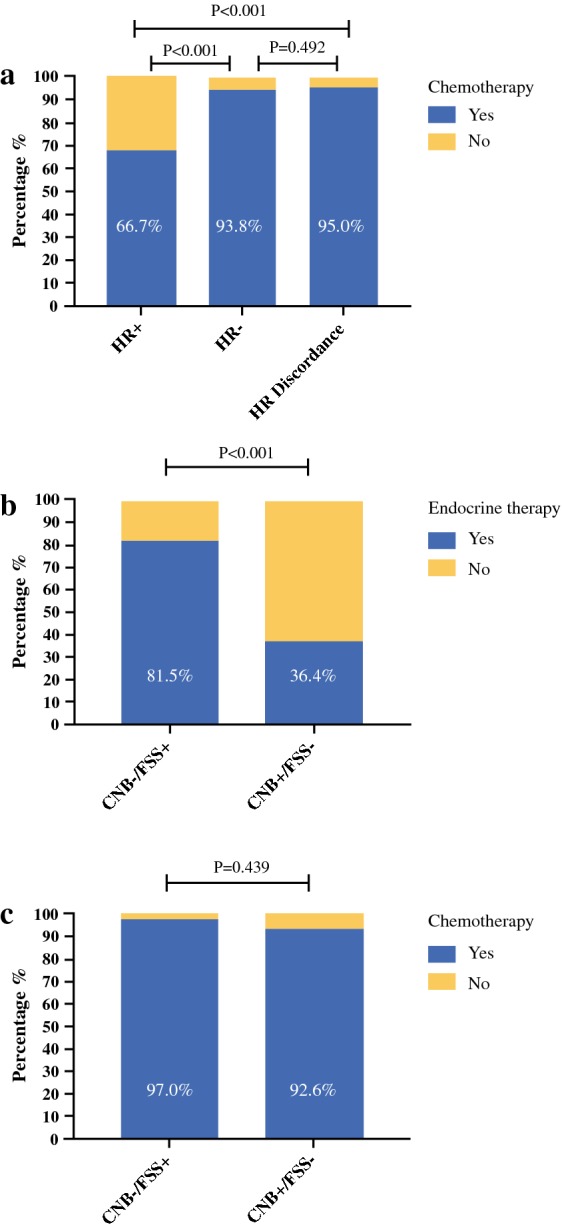
Hormonal receptor discordance and adjuvant systemic therapy: **a** Usage of chemotherapy according to hormonal receptor status between CNB and FSS. The rates of chemotherapy were statistically different among three groups (*P *< 0.001). A total of 57 (95.0%) patients with HR discordance tumors were given adjuvant chemotherapy, higher than among patients in the both HR positive group (66.7%, *P* < 0.001); **b** Rate of endocrine therapy in patients with CNB−/FSS+ and CNB+/FSS− tumors; **c** Rate of chemotherapy in patients with CNB−/FSS+ and CNB+/FSS− tumors

Adjuvant therapy decisions were made by multidisciplinary discussion meeting in all patients. There were 313 (18.3%) patients who received 21-gene recurrence score testing for adjuvant chemotherapy decision-making. A total of 57 (95.0%) patients with HR discordance tumors were given adjuvant chemotherapy, similar to patients in the HR negative group (93.8%, *P* = 0.492) but higher than patients in the HR positive group (66.7%, *P* < 0.001, Fig. 2a). There were only 34 patients (56.7%) in the HR discordance group who received adjuvant endocrine therapy (Fig. 2b). Among patients with HR CNB−/FSS+ tumors, 22 (81.5%) received endocrine therapy, which was higher than among those with HR CNB+/FSS− tumors (36.4%, 12/33) (*P* < 0.001). However, there was no chemotherapy usage rate difference between the HR CNB−/FSS+ and HR CNB+/FSS− groups (97.0% versus 92.6%, respectively, *P* = 0.439, Fig. 2c).

### Hormonal Receptor Discordance and Clinical Outcomes

There were three patients lost to follow-up and not included for survival analysis. After median follow-up of 34.8 months (range 4.2–75.8 months), 140 patients had disease relapse. HR status was associated with DFS in the whole population (*P* = 0.009). The estimated 5-year DFS rate was 87.0% (95% CI 83.9–90.1%), 83.2% (95% CI 81.1–85.3%), and 90.4% (95% CI 85.42–95.38%) among patients in the HR positive, HR negative, and HR discordance groups, respectively (Supplementary Fig. 2a). Patients with both HR negative tumors had worse DFS than those in the HR positive group (*P* = 0.002). However, patients in the HR discordance group had similar DFS compared with patients in the HR positive (*P* = 0.653) or HR negative (*P* = 0.522) groups (Supplementary Fig. 2a). A total of 48 patients died during follow-up. The 5-year OS rates were 94.8, 94.3, and 93.2% in the HR positive, HR negative, and HR discordance groups, respectively (*P* = 0.292) (Supplementary Fig. 2b).

Univariate analysis found that tumor size, lymph node status, HR status of FSS, Ki67 level, HER2 status, and HR discordant status were associated with DFS (Supplementary Table 3, *P* < 0.05). Multivariable analysis demonstrated that tumor size, lymph node status, HR status, and Ki67 level were independently associated with DFS (Supplementary Table 4, *P* < 0.05).

Among patients with discordance HR tumors, the 5-year DFS was 94.4% and 87.3% in the HR CNB−/FSS+ and CNB+/FSS− groups (*P* = 0.966, Supplementary Fig. 2c). In addition, there was no OS difference between the HR CNB−/FSS+ and CNB+/FSS− groups (94.7% versus 90.9%, *P* = 0.882, Supplementary Fig. 2d). Furthermore, among the 60 patients with HR discordance tumors, there was no DFS (*P* = 0.259) or OS (*P* = 0.508) difference between patients received endocrine therapy or not.

## Discussion

Breast cancer has been identified as having at least four subtypes: luminal-like, HER2-positive, basal-like, and normal-like.^11^,^12^ In clinical practice, IHC results on ER, PgR, HER2, and Ki67 status can be used to approximately classify breast cancer into the above subtypes.^13^ CNB is widely used in BC diagnosis and ER, PR, HER2, and Ki67 status evaluation. However, due to smaller sample size and tumor heterogeneity, biomarker testing in CNB may not be reliable compared with in FSS. The current study included 1710 patients, nearly 30% of whom received breast-conserving surgery, relatively lower than the rate in the USA but much higher than the average rate in China.^14^ All patients had paired CNB and FSS to test ER, PR, HER2, and Ki67. To the best of the authors’ knowledge, this study enrolled the largest number of breast cancer patients with paired CNB and FSS samples to evaluate ER, PR, HER2, and Ki67 status, finding that invasive BC patients with discordance HR status had similar tumor characteristics, adjuvant chemotherapy usage, and disease outcome compared with patients with both HR negative tumors.

The current study reveals good agreement in HR and HER2 status evaluation between CNB and FSS, whereas the Ki67 expression level was slightly higher in FSS samples. The concordance rate for HR status testing was 96.5% in this study, indicating a good correlation between CNB and FSS, similar to other studies.^15^ The ER concordance rate was relatively higher than that for PgR, and some other studies also found that the rate of HR positivity was higher in CNB samples than FSS specimens,^16^,^17^ which can likely be explained by the poorer fixation of FSS compared with CNB specimens, including delayed fixation, underfixation, and overfixation with formalin prior to IHC analysis.^9^,^15^ Another reason may be tumor heterogeneity, with the core biopsy not reflecting the status of the entire tumor.^18^ However, the results of this study did not show that larger tumor was associated with higher discordance rate.

The ASCO/CAP guideline recommended 1% as the cutoff value for ER or PgR positivity, leading to more patients receiving adjuvant endocrine therapy.^9^ In this study, there were 60 patients (3.5%) with discordance HR status between CNB and FSS, which were further classified as HR CNB+/FSS− and CNB−/FSS+. Among these patients, most of the tumors expressed low level of ER positivity. More importantly, these HR discordance patients had similar tumor characteristics to HR negative patients, who had higher Ki67 expression and more HER2 positive tumors than patients in the HR positive group. These discrepancies would further influence the usage of adjuvant chemotherapy, and there were only three cases in the HR discordance group who did not receive adjuvant chemotherapy. The rate of adjuvant endocrine therapy was 56.7% in the HR discordance group, and even lower in the HR CNB+/FSS− subgroup (36.4%), indicating that physicians prefer to treat these HR discordant patients as HR negative disease, i.e., with more adjuvant chemotherapy and less endocrine therapy.

To the best of the authors’ knowledge, the current study includes the largest cohort to date used to investigate the impact of HR discordance on disease outcome in breast cancer patients. Patients with HR discordance tumor had similar disease outcome compared with HR negative or HR positive group. In addition, among HR discordance patients, we found that adjuvant endocrine therapy did not improve disease outcome, which may be explained by the 31 patients (51.7%) with low ER expression level among these patients. Although the HR discordant group was relatively small, which might be a limitation to make conclusions, the results of this study show that the survival curves between CNB+/FSS− and CNB−/FSS+ were very close (*P* = 0.966). According to the ASCO/CAP guideline, HR positivity was defined as more than 1% tumor cell with nuclear staining. However, the 2015 St. Gallen International Expert Consensus recommended that ER expression values between 1% and 9% be considered as equivocal with uncertain benefit of endocrine therapy.^13^ Moreover, data from MDACC showed that patients with 1–9% ER positivity clinically behaved like those with HR negative breast cancer in terms of pCR and survival outcomes. Furthermore, low ER expression patients received limited benefit from adjuvant hormonal therapy, indicating that the ER positivity cutoff should be redefined as 10%, to better predict the treatment response and disease outcome.^19^ In addition, these results may support the use of ablative therapy in breast cancer, which has often been challenged due to the lack of pathological examination of the final surgical specimen. Our results show that additional HR testing on FSS may not guide treatment selection or improve patient outcome.

Besides ER and HR status evaluation, this study also shows good concordance for HER2 testing in CNB samples. However, Ki67 testing in CNB was not as accurate as ER or HER2 evaluation. Ki67 expression level was much higher in FSS samples, which may be caused by tumor heterogeneity and wound response after biopsy. Ki67 has been recommended as a key clinicopathological marker to distinguish luminal A and luminal B tumors since 2009.^20^ However, there is a consistent debate about standardization of Ki67 pathological interpretation and the optimal cutoff value for high Ki67 expression.^21^,^22^

This study has several limitations, which should be considered when interpreting these results. Firstly, this was a retrospective study, and treatment was not randomly assigned. The reason for a lack of survival difference between the HR discordance group and the other two groups may be a result of treatment selection bias and the relatively small numbers of patients enrolled. However, given the small number of patients, conducting a randomized controlled trial that included patients with HR discordance tumors would be extremely difficult. Secondly, the follow-up time is slightly short for survival analysis, which may influence the results. Longer follow-up time will guarantee the reliability of our findings. Last but not least, comparison of the messenger RNA (mRNA) level of ER between these three groups is lacking, which might better elucidate the real cause of the difference in tumor characteristics among these groups.

In conclusion, the results of this study show a high concordance rate and good agreement between CNB and FSS for ER, PgR, HR, and HER2 evaluation. The small percentage of patients with discordance HR status between CNB and FSS had similar tumor characteristics, adjuvant chemotherapy usage, and disease outcome compared with patients in the HR negative group. Patients in the HR discordance group received little benefit from adjuvant endocrine therapy, which deserves further clinical evaluation.

## Electronic supplementary material

Below is the link to the electronic supplementary material.

Supplementary material 1 (DOCX 41 kb)

Supplementary Fig. 1 ER and PgR expression level of 60 patients with discordant HR status between CNB and FSS. (X-axis: Number of case with HR discordance tumor (1-60); Y-axis: Percentage of ER/PgR expression of those patients) a). ER expression level of 60 HR discordant patients. There were 31 patients with low ER expression less than 10%: 14 with CNB-/FSS+ and 17 with CNB+/FSS-. b). PgR expression level of 60 HR discordant patients. Abbreviation: CNB, Core needle biopsy; FSS, Following surgical samples; ER, Estrogen receptor; PgR, Progesterone receptor; HR, hormonal receptor (JPEG 367 kb)

Supplementary Fig. 2 The Kaplan–Meier analysis for DFS and OS according to hormonal receptor (HR) status between CNB and FSS. a) DFS according to HR status between CNB and FSS (P=0.009). The estimated 5-year DFS rates were 87.0%, 83.2%, and 90.4% among patients with both HR positive, both HR negative, and HR discordance tumors. b) OS according to HR status between CNB and FSS. The 5-year OS rates were 94.8%, 94.3% and 93.2%, respectively, in patients with both HR positive, both HR negative, and HR discordance. (P=0.292). c). DFS in patients with discordant HR tumors. The 5-year DFS was 94.4% and 87.3% in the CNB-/FSS+ and CNB+/FSS- group (P=0.966). d) OS in patients with discordant HR tumors. The 5-year OS was 94.7% and 90.9% in the CNB-/FSS+ and CNB+/FSS- group (P=0.822) (JPEG 176 kb)
