# Supplementary material for: Clinicopathological Features and Disease Outcome in Breast Cancer Patients with Hormonal Receptor Discordance between Core Needle Biopsy and Following Surgical Sample
Source: Ann Surg Oncol. 2019 May 29;26(9):2779–86. doi: 10.1245/s10434-019-07480-y (PMC6682563; doi:10.1245/s10434-019-07480-y)
Supplement: Supplementary file 1 — Supplementary material 1 (DOCX 41 kb) [file 10434_2019_7480_MOESM1_ESM.docx]

Supplementary table 1

Tumor characteristics for CNB and FSS results

| Characteristics | | CNB | No. (%) | |  | | FSS | *P* |
| --- | --- | --- | --- | --- | --- | --- | --- | --- |
| Pathological type | | |  | |  | |  | 0.738 |
| IDC  ILC  Others | | 1555(90.9)  40(2.3)  115(6.7) |  | | 1546(90.4)  47(2.7)  117(6.8) | | |  |
| Histological grade | | |  |  | |  | | <0.001 |
| I  II  III  NA | | 69(4.0)  843(49.3)  615(36.0)  183(10.7) |  | | 41(2.4)  748(43.8)  777(45.5)  143(8.4) | | |  |
| HR |  |  |  |  | |  | | 0.846 |
| Positive  Negative | | 1266(74.0)  444(26.0) |  | | 1260(73.7)  450(26.3) | | |  |
| ER |  |  |  |  | |  | | 0.698 |
| Positive  Negative | | 1262(73.8)  448(26.2) |  | | 1252(73.2)  458(26.8) | | |  |
| PgR | | |  |  | |  | | 0.085 |
| Positive  Negative | | 940(55.0)  770(45.0) |  | | 990(57.9)  720(42.1) | | |  |
| HER2 | |  |  |  | |  | | 0.064 |
| Positive  Negative | | 336(19.6)  1374(80.4) |  | | 380(22.2)  1330(77.8) | | |  |
| Ki67(％) | |  |  |  | |  | | <0.001 |
| Mean | | 24.3 |  | | 28.6 | | |  |
| Ki67 | | |  | |  | | | <0.001 |
| <14%  ≥14% | | 763(44.6)  947(55.4) |  | | 565(33.0)  1145(67.0) | | |  |

*CNB* Core needle biopsy, *FSS* Following surgical samples, *IDC* Invasive ductal carcinoma, *ILC* Invasive lobular carcinoma, *HR* Hormonal receptor, *ER* Estrogen receptor, *PgR* Progesteron receptor, *HER2* Human epidermal growth factor receptor-2

Supplementary table 2

Concordance between CNB and FSS for receptor status and Ki67

| CNB | | FSS  Positive Negative | | | Concordance  rate | | | Kappa | *P* |
| --- | --- | --- | --- | --- | --- | --- | --- | --- | --- |
| ER | |  |  |  |  | 96.5% |  | 0.910 | <0.001 |
| Positive  Negative | | 1227  25 |  | 35  423 |  | | |  |  |
| PgR | | | |  |  | 91.1% |  | 0.819 | <0.001 |
| Positive  Negative | | 889  101 |  | 51  669 |  | | |  |  |
| HR | |  |  |  |  | 96.5% |  | 0.909 | <0.001 |
| Positive  Negative | | 1233  27 |  | 33  417 |  | | |  |  |
| HER2 | |  |  |  |  | 95.3% |  | 0.859 | <0.001 |
| Positive  Negative | | 318  62 |  | 18  1312 |  | | |  |  |
| Ki67 |  | <14% | | ≥14% |  | 81.5% |  | 0.616 | <0.001 |
| <14%  ≥14% | | 506  59 |  | 257  888 |  | | |  |  |

*CNB* Core needle biopsy, *FSS* Following surgical samples, *HR* Hormonal receptor, *HER2* Human epidermal growth factor receptor-2, *ER* Estrogen receptor, *PgR* Progesterone receptor

Supplementary table 3

Univariate analyses of DFS and OS according to tumor characteristics

| Factor |  | DFS  *P* | OS  *P* |
| --- | --- | --- | --- |
| Age |  | 0.741 | 0.899 |
| Menstrual status |  | 0.348 | 0.437 |
| Pathological type |  | 0.939 | 0.355 |
| Histological grade |  | 0.142 | 0.260 |
| Tumor size |  | <0.001 | 0.029 |
| Axillary lymph node |  | <0.001 | 0.013 |
| HR status(FSS) |  | 0.003 | 0.139 |
| Ki67 (FSS) |  | 0.003 | 0.013 |
| HER2 status(FSS) |  | 0.03 | 0.283 |
| HR discordant status |  | 0.013 | 0.319 |
| Chemotherapy |  | 0.600 | 0.720 |

*DFS* Disease-free survival, *FSS* Following surgical samples, *OS* Overall survival, *HR* Hormonal receptor, *HER2* Human epidermal growth factor receptor-2

Supplementary table 4

Univariate and multivariate analyses of DFS according to tumor characteristics

| Factor | N(%) | DFS  Univariate  *P HR(95%CI)* | Multivariate  *P* *HR*(95％CI) |
| --- | --- | --- | --- |
| Age |  | 0.741 | / |
| ≤50  >50 | 559(32.7)  1151(67.3) | 1  0.741 0.942(0.661-1.342) |  |
| Menstrual status |  | 0.348 | / |
| Peri/pre-menopause  Post-menopause | 597(34.9)  1113(65.1) | 1  0.348 1.189(0.828-1.708) |  |
| Pathological type |  | 0.939 | / |
| IDC  ILC  Others | 1546(90.4)  47(2.7)  117(6.8) | 1  0.837 0.887(0.282-2.789)  0.778 1.093(0.590-2.024) |  |
| Histological grade |  | 0.142 | / |
| I  II  III  NA | 41(2.4)  748(43.8)  777(45.5)  143(8.4) | 1  0.389 0.407(0.052-3.152)  0.583 0.834(0.435-1.597)  NS |  |
| Tumor size |  | <0.001 | 0.005 |
| ≤2cm  >2cm  NA | 857(50.1)  848(49.6)  5(0.3) | 1  <0.001 2.025(1.427-2.873)  NS | 1  0.005 1.674（1.172-2.391）  NS |
| Axillary lymph node |  | <0.001 | <0.001 |
| Negative  Positive  NA | 1003(58.7)  702(41.1)  5(0.3) | 1  <0.001 2.044(1.456-2.866)  NS | 1  <0.001 1.883（1.336-2.655）  NS |
| HR Status(FSS) |  | 0.003 | 0.028 |
| Positive  Negative | 1260(73.7)  450(26.3 | 1  0.003 1.696(1.199-2.398) | 1  0.028 1.513（1.045-2.191） |
| Ki67(FSS) |  | 0.003 | 0.049 |
| <14%  ≥14% | 565(33.0)  1145(67.0) | 1  0.003 1.865(1.245-2.795) | 1  0.049 1.520（1.001-2.307） |
| HER2 Status(FSS) |  | 0.03 | 0.771 |
| Negative  Positive | 1330(77.8)  380(22.2) | 1  0.03 1.504(1.041-2.171) | 1  0.771 1.061(0.714-1.576) |
| HR discordant Status |  | 0.013 | 0.075 |
| Positive  Negative  HR Discordance | 1233(72.1)417(24.4)  60(3.5) | 1  0.002 1.733(1.215-2.472)  0.647 1.235(0.501-3.042) | 1  0.025 1.544(1.056-2.256) 0.949 1.031(0.410-2.591) |
| Chemotherapy |  | 0.600 | / |
| No  Yes | 438(25.6)  1272(74.4) | 1  0.600 1.110(0.751-1.641) |  |

*Uv* univariate, *Mv* multivariate, *DFS* Disease-free survival, *OS* Overall survival, *HR* Hormonal receptor, *HER2* Human epidermal growth factor receptor-2, *IDC* Invasive ductal carcinoma, *ILC* Invasive lobular carcinoma

Supplementary table 5

Univariate and multivariate analyses of OS according to tumor characteristics

| Factor | N(%) | OS  Uv  *P* *HR*(95％CI) | Mv  *P* *HR*(95%CI) |
| --- | --- | --- | --- |
| Age |  | 0.899 | / |
| ≤50  >50 | 559(32.7)  1151(67.3) | 1  0.899 1.041(0.562-1.926) |  |
| Menstrual status |  | 0.437 | / |
| Peri/pre-menopause  Post-menopause | 597(34.9)  1113(65.1) | 1  0.437 1.282(0.685-2.401) |  |
| Pathological type |  | 0.355 | / |
| IDC  ILC  Others | 1546(90.4)  47(2.7)  117(6.8) | 1  0.487 1.654(0.401-6.826)  0.216 0.286(0.039-20.78) |  |
| Histological grade |  | 0.260 | / |
| I  II  III  NA | 41(2.4)  748(43.8)  777(45.5)  143(8.4) | 1  0.473 2.410(0.218-26.629)  0.646 1.409(0.326-6.097)  0.228 2.419(0.575-10.176) |  |
| Tumor size |  | 0.029 | 0.145 |
| ≤2cm  >2cm  N/A | 857(50.1)  848(49.6)  5(0.3) | 1  0.033 1.908(1.055-3.449)  NS | 1  0.145 1.566(0.856-2.863)  NS |
| Axillary lymph node |  | 0.013 | 0.042 |
| Negative  Positive  NA | 1003(58.7)  702(41.1)  5(0.3) | 1  0.014 2.061(1.154-3.680)  NS | 1  0.042 1.842(1.024-3.315)  NS |
| HR Status(FSS) |  | 0.139 | / |
| Positive  Negative | 1260(73.7)  450(26.3 | 1  0.139 1.575（0.863-2.875） |  |
| Ki67(FSS) |  | 0.013 | 0.026 |
| <14%  ≥14% | 565(33.0)  1145(67.0) | 1  0.013 2.610（1.221-5.579） | 1  0.026 2.378(1.108-5.102) |
| HER2 Status(FSS) |  | 0.283 | / |
| Negative  Positive | 1330(77.8)  380(22.2) | 1  0.283 1.418(0.750-2.682) |  |
| HR discordant Status |  | 0.319 | / |
| Positive  Negative  HR Discordance | 1233(72.1)417(24.4)  60(3.5) | 1  0.562 1.528(0.365-6.391)  0.132 1.608(0.867-2.982 ) |  |
| Chemotherapy |  | 0.720 | / |
| No  Yes | 438(25.6)  1272(74.4) | 1  0.720 0.889(0.469-1.686) |  |

*Uv* univariate, *Mv* multivariate, *DFS* Disease-free survival, *OS* Overall survival, *HR* Hormonal receptor, *HER2* Human epidermal growth factor receptor-2, *IDC* Invasive ductal carcinoma, *ILC* Invasive lobular carcinoma
