# Supplementary figures and images for: Clinicopathological Features and Disease Outcome in Breast Cancer Patients with Hormonal Receptor Discordance between Core Needle Biopsy and Following Surgical Sample
Source: Ann Surg Oncol. 2019 May 29;26(9):2779–86. doi: 10.1245/s10434-019-07480-y (PMC6682563; doi:10.1245/s10434-019-07480-y)

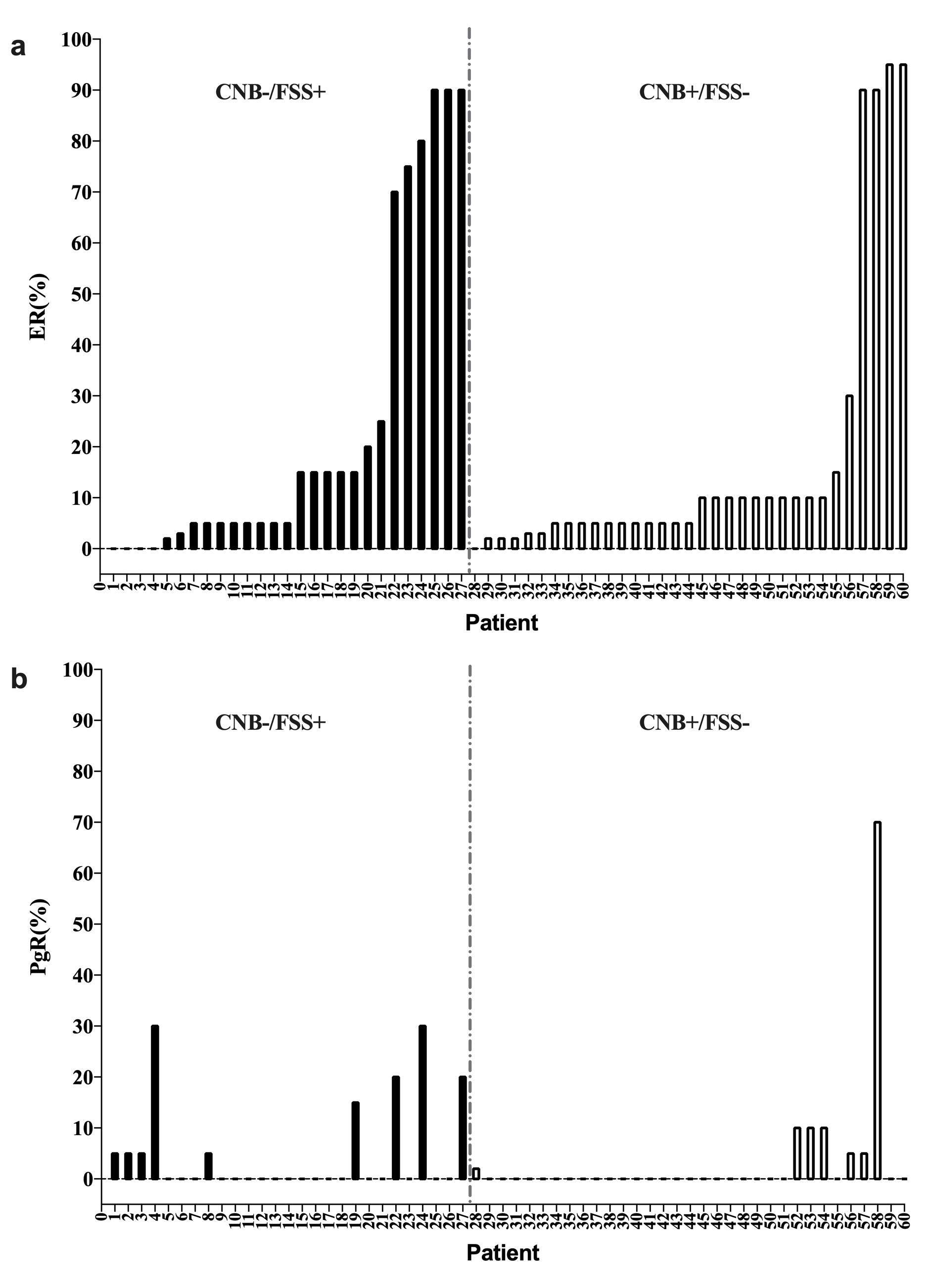

Supplement: Supplementary file 2 — ER and PgR expression level of 60 patients with discordant HR status between CNB and FSS. (X-axis: Number of case with HR discordance tumor (1-60); Y-axis: Percentage of ER/PgR expression of those patients) a). ER expression level of 60 HR discordant patients. There were 31 patients with low ER expression less than 10%: 14 with CNB-/FSS+ and 17 with CNB+/FSS-. b). PgR expression level of 60 HR discordant patients. Abbreviation: CNB, Core needle biopsy; FSS, Following surgical samples; ER, Estrogen receptor; PgR, Progesterone receptor; HR, hormonal receptor (JPEG 367 kb) [file 10434_2019_7480_MOESM2_ESM.jpg]

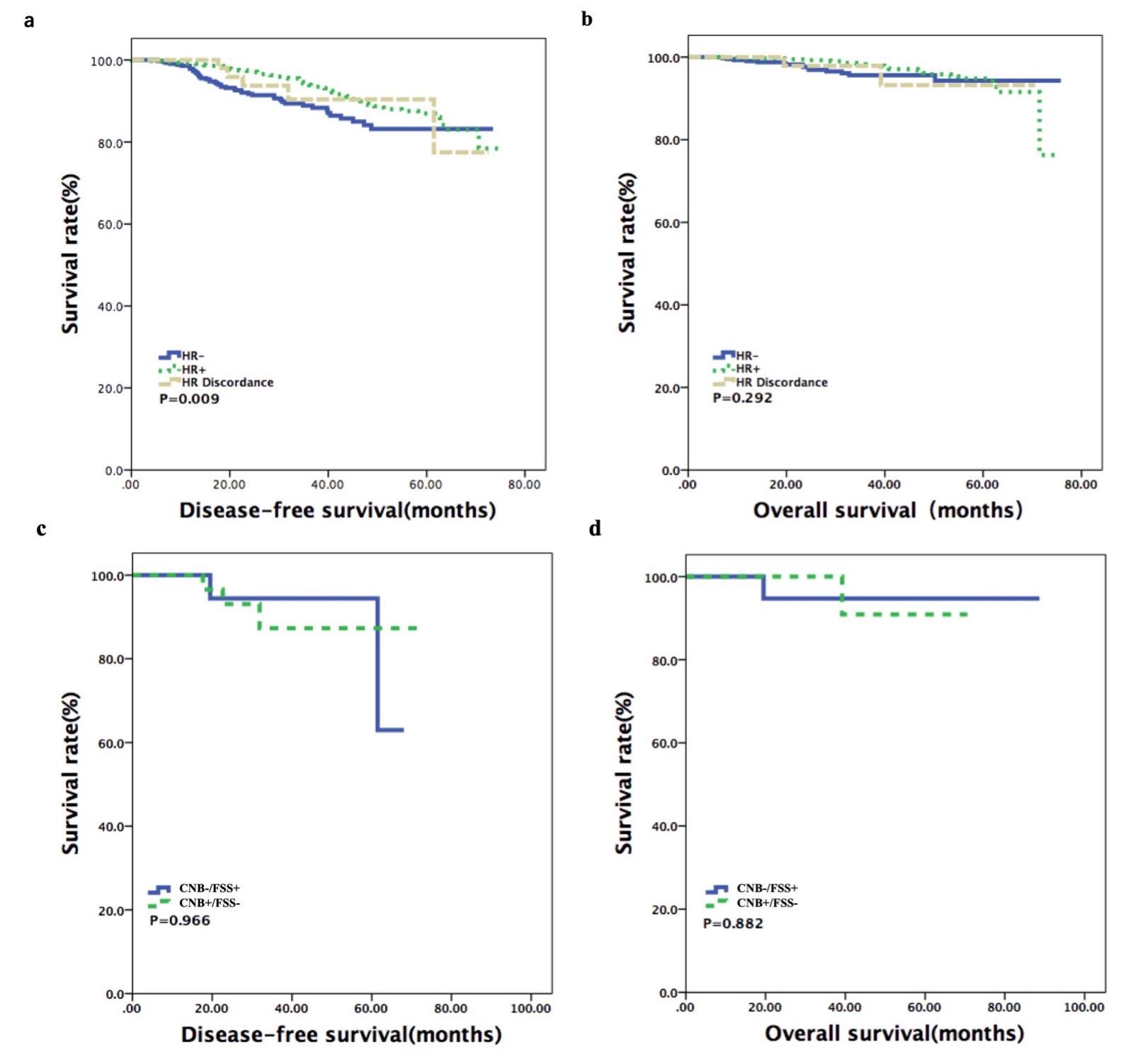

Supplement: Supplementary file 3 — The Kaplan–Meier analysis for DFS and OS according to hormonal receptor (HR) status between CNB and FSS. a) DFS according to HR status between CNB and FSS (P=0.009). The estimated 5-year DFS rates were 87.0%, 83.2%, and 90.4% among patients with both HR positive, both HR negative, and HR discordance tumors. b) OS according to HR status between CNB and FSS. The 5-year OS rates were 94.8%, 94.3% and 93.2%, respectively, in patients with both HR positive, both HR negative, and HR discordance. (P=0.292). c). DFS in patients with discordant HR tumors. The 5-year DFS was 94.4% and 87.3% in the CNB-/FSS+ and CNB+/FSS- group (P=0.966). d) OS in patients with discordant HR tumors. The 5-year OS was 94.7% and 90.9% in the CNB-/FSS+ and CNB+/FSS- group (P=0.822) (JPEG 176 kb) [file 10434_2019_7480_MOESM3_ESM.jpg]
